# Supplementary material for: Multi-Temperatures Pyrolysis Gas Chromatography: A Rapid Method to Differentiate Microorganisms
Source: Microorganisms. 2022 Nov 25;10(12):2333. doi: 10.3390/microorganisms10122333 (PMC9781292; doi:10.3390/microorganisms10122333)
Supplement: Supplementary file 1 [file microorganisms-10-02333-s001.zip › microorganisms-1944562-supplementary.pdf]

Multi-temperatures pyrolysis gas chromatography: A rapid method to differentiate microorganisms

Yun Yang Wan*, Ying Jia Zhu, Liang Jiang, Na Luo

State Key Laboratory of Petroleum Resources and Prospecting, Research Centre for Geomicrobial Resources and Application, Unconventional Petroleum Research Institute, College of Geosciences, China University of Petroleum, Beijing 102249, China

Supplementary materials

Funds: PetroChina Major Strategic Cooperation Projects (ZLZX2020010805, ZLZX2020020405), Forward Looking Foundation of China University of Petroleum-Beijing (ZX20190209), the National Natural Science Foundation of China (41373086, 41373126), National Major Scientific and Technological Special Projects (No. 2016ZX05050011, 2016ZX05040002), Beijing Nova Program and Leading Talent Culturing Cooperative Projects (Z161100004916033)

***Corresponding author:** Tel: 86-010-89732230; E-mail: wanyunyang@cup.edu.cn

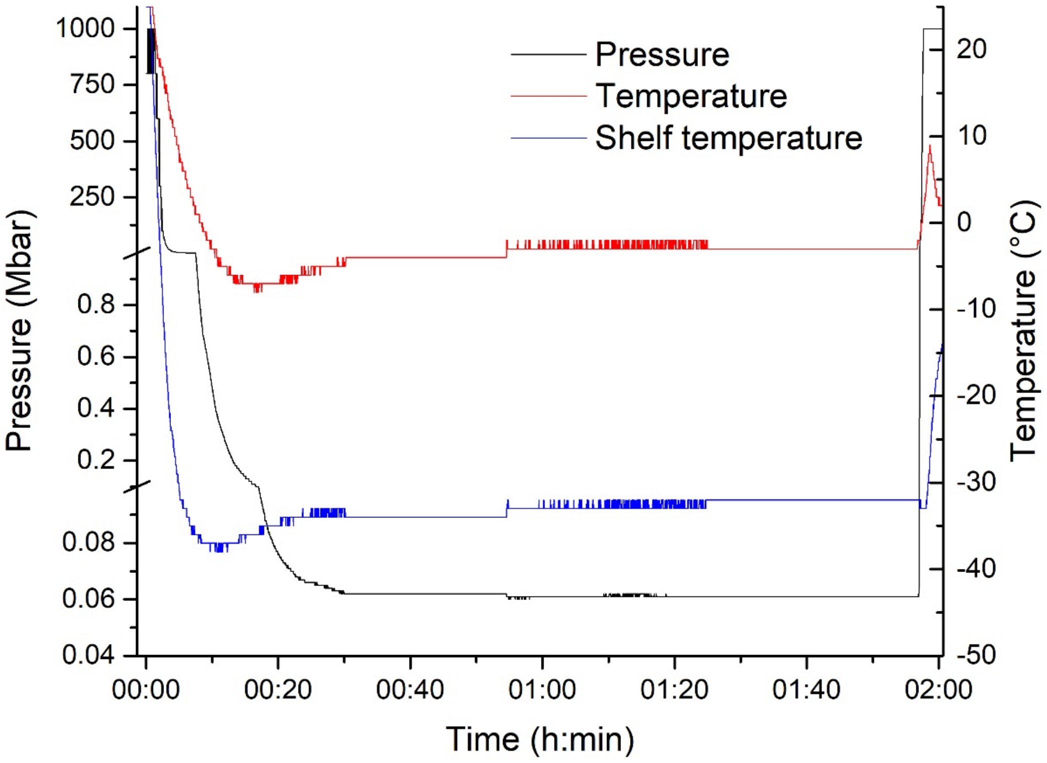

Figure S1 Vacuum freeze-drying curves of sample preparation.

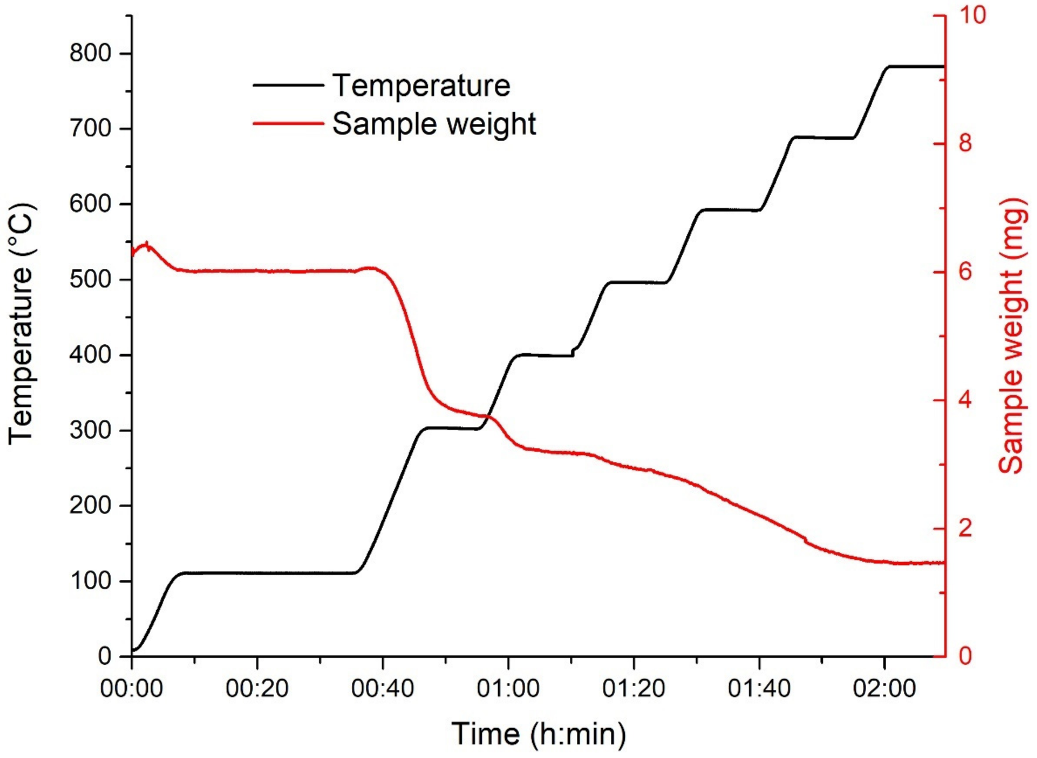

Figure S2 Thermogravimetric analysis of *E.coli.*

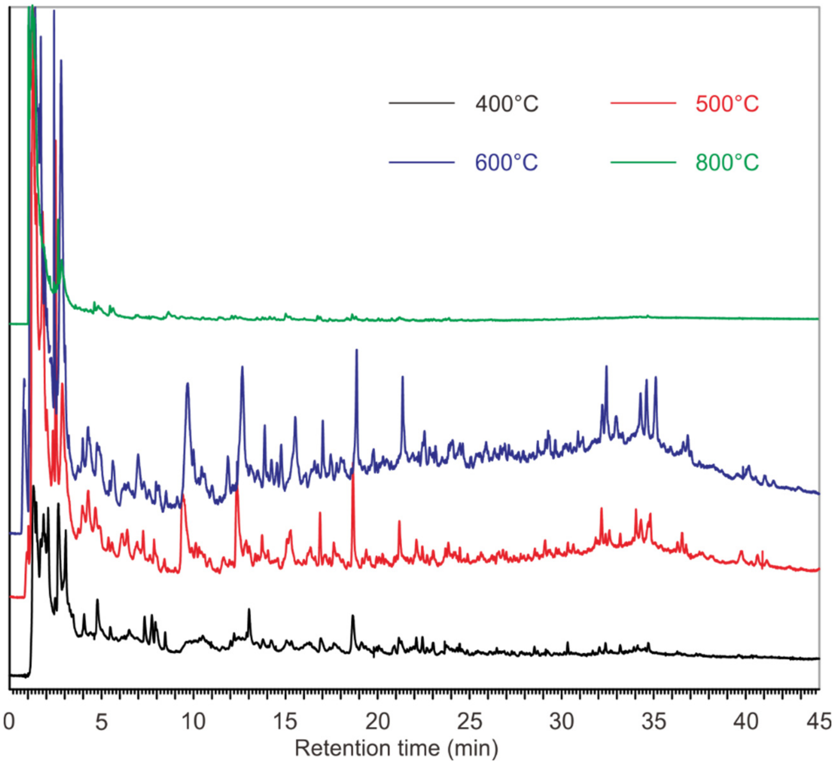

Figure S3 Pyrograms of *P. coccineus* at different temperatures.

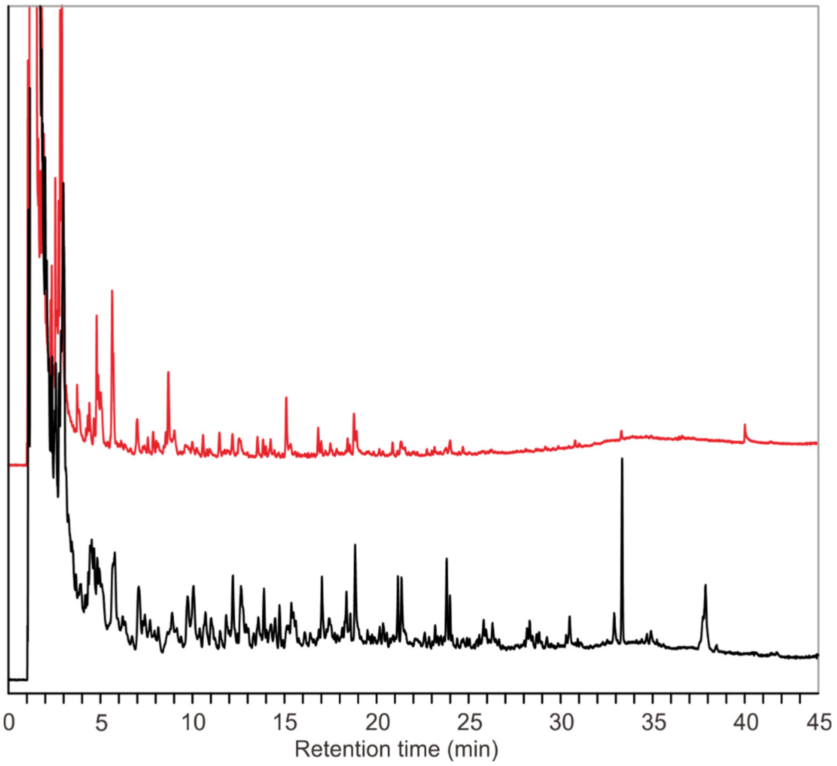

Figure S4 Pyrograms of *S. bovis* at 600 °C (black) and 800 °C (red).

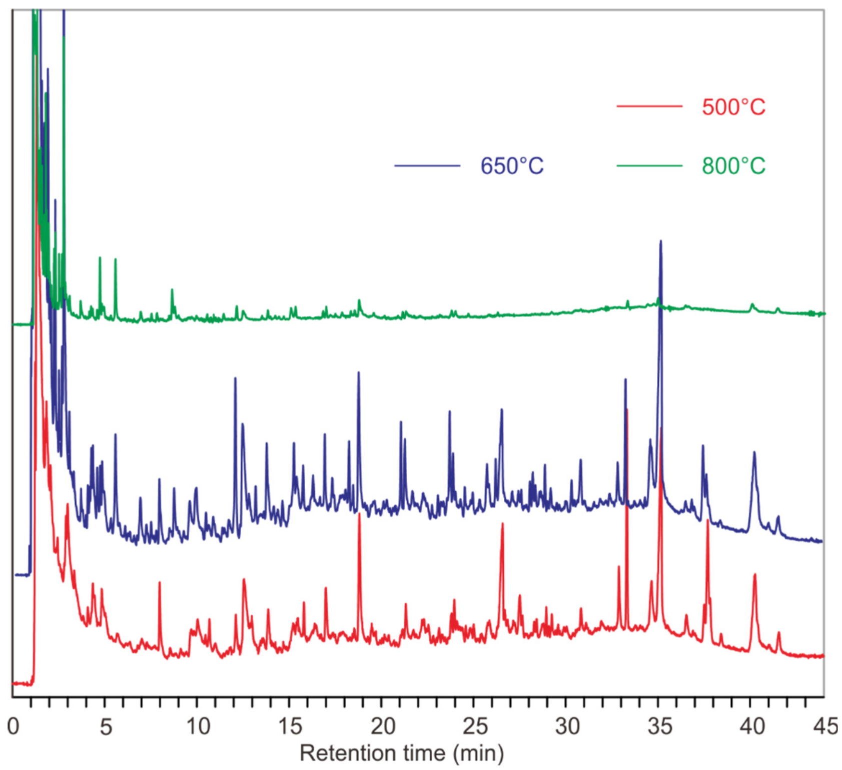

Figure S5 Pyrograms of *A. calcoaceticus* at three different temperatures.

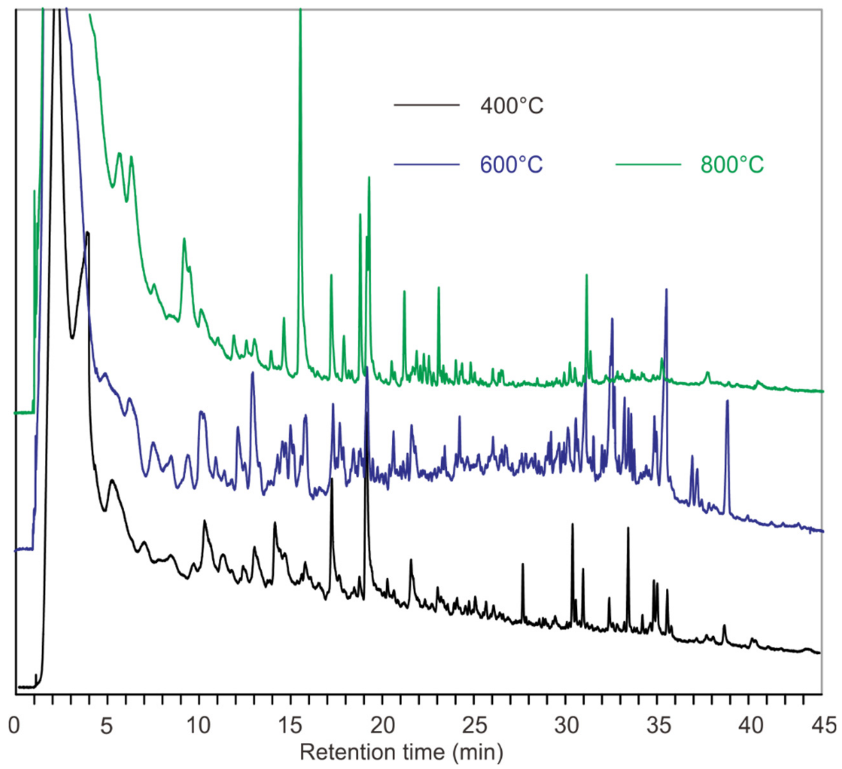

Figure S6 Pyrograms of *C. oxamicus* at three different temperatures.
